# Factors in Placement and Enrollment of Primary Care Patients in YMCA’s Diabetes Prevention Program, Bronx, New York, 2010–2015

**DOI:** 10.5888/pcd14.160486

**Published:** 2017-03-30

**Authors:** Earle C. Chambers, Colin D. Rehm, Jordan Correra, Lydia Elena Garcia, Melinda E. Marquez, Judith Wylie-Rosett, Amanda Parsons

**Affiliations:** 1 Department of Family and Social Medicine, Albert Einstein College of Medicine, Bronx, New York; 2 Office of Community and Population Health, Montefiore Health System, Bronx, New York; 3 Department of Epidemiology and Population Health, Albert Einstein College of Medicine, Bronx, New York; 4 YMCA of Greater New York, New York, New York

## Abstract

**Introduction:**

The reach of the New York State YMCA’s Diabetes Prevention Program (DPP) to at-risk populations may be increased through integration with primary care settings. Although considerable effort has been made in the referral and retention of patients, little is known about the factors associated with the placement of potential participants into YMCA’s DPP.

**Methods:**

Among Montefiore Health System (MHS) patients referred to YMCA’s DPP (n = 1,249) from July 10, 2010, through November 11, 2015, we identified demographic factors (eg, age, preferred language) and primary care practice-level factors (eg, time between referral and start of session, session season) associated with placement into a session and subsequent drop-out. We also evaluated factors associated with weight loss.

**Results:**

Patients were predominantly female (71%) and aged 45 years or older (71%). Patients preferring sessions in Spanish were less often placed in sessions. Patients aged 18 to 44 years were less often placed (*P* = .01) and enrolled (*P* = .001) than patients aged 60 years or older. Sessions conducted in the summer and spring had higher enrollment than fall and winter months. Patients who started the YMCA’s DPP within 2 months of their referral date were more often enrolled (54.4%) than patients who waited 4 or more months (21.6%) to start their sessions. Patients aged 45 to 59 years lost marginally less weight than those aged 60 years or older (−3.1% vs −3.8%; *P* = .07).

**Conclusion:**

Although this evaluation gives some insight into the barriers to placement and enrollment in YMCA’s DPP, challenges remain. Efforts are under way to increase referral of patients to community-based DPPs.

## Introduction

The Centers for Disease Control and Prevention (CDC) estimates that 35% of US adults have prediabetes (1). Approximately 11% of people with prediabetes will develop overt diabetes each year without any intervention (2). CDC’s National Diabetes Prevention Program (National DPP) (3,4) lifestyle modification intervention is an evidence-based approach to reducing the risk of developing type 2 diabetes among individuals with prediabetes. The Diabetes Prevention Program trial showed that, relative to no intervention, the lifestyle intervention promoting a healthful diet and increased physical activity decreased risk of developing diabetes after 10 years of follow-up by 34% (5).

The YMCA has adapted the National DPP model and offers it among its wellness programs. The 2010 through 2012 results from the New York State YMCA’s Diabetes Prevention Program (YMCA’s DPP) from 14 sites showed average weight loss approaching the 5% weight loss reported in the DPP randomized trial; 40% reached 5% weight loss and average weight loss was 4.2% (6). Although participants in the YMCA’s DPP were successful at reaching weight-loss goals, participants were predominantly white and female, and only 6.7% received Medicaid compared with 27% in New York State overall. Further analysis of the YMCA’s DPP showed that black participants as well as those with less education or with lower income were less likely to complete the program. Among the recommendations of this demonstration project was a “need for targeted approaches to reach and retain a broader population, including men, minorities, and individuals who are low-income and uninsured or publicly insured” (6,7).

Ackermann et al reported that 24% of participants placed in YMCA’s DPP did not attend any sessions at all, and 15% were lost to follow-up by months 4 through 6 (8). Within the clinical setting, factors associated with the referral of patients to a DPP by the health care provider and factors associated with attrition among patients currently enrolled in a DPP are the focus of much of the engagement strategies regarding DPPs (9). It is unclear what happens between the point of referral from the health care provider to the YMCA’s DPP and subsequent progression of the patient through the program. Our study examined the demographic and primary care practice–related factors associated with the placement (patients scheduled to participate) and enrollment (patients completing ≥3 sessions) of patients referred to YMCA’s DPP. Furthermore, we examined the effect of these same factors on weight loss.

## Methods

### Program overview

Montefiore Health System (MHS) is a large integrated health system in the Bronx and Hudson Valley serving roughly 85% government payer (Medicaid and/or Medicare) patients. Beginning in 2010, MHS partnered with the YMCA of Greater New York to provide the 1-year YMCA’s DPP to eligible patients visiting Bronx-based primary-care clinics. Eligibility was based on criteria established by the YMCA and CDC, which were being aged 18 years or older, having no previous diabetes diagnosis (excluding gestational diabetes), being overweight or obese (body mass index [BMI] ≥25; ≥22 if Asian), and having a hemoglobin A_1c_ between 5.7% and 6.4% (or fasting plasma glucose 100–125 mg/dL or 2-hour plasma glucose 140–199 mg/dL).

During an office visit, eligible patients were told by their physician of their risk of developing diabetes and were asked if they were interested in participating in the YMCA’s DPP. If the patient expressed interest, the physician referred the patient to the YMCA using the referral order in the electronic health record (EHR) system. The EHR system generated a referral form populated with the patient’s demographic and BMI information, which was faxed to the YMCA’s DPP after the referring physician obtained consent from the patient. The YMCA then entered patient, physician, and practice information into its internal database and attempted to contact the patient for placement. The schedule and location for starting new groups for the 16 core sessions of the program were based on the availability of lifestyle coaches, space to hold sessions, and patient demand. Attempts were made to place patients in the YMCA’s core groups for up to 1 year after the referral. There were 66 core groups in which MHS patients were placed over the study period. The program was made available to all patients at no charge because of in-kind donations from the YMCA of Greater New York and other grant-based funding. The YMCA’s DPP sessions were offered by trained lifestyle coaches in English or Spanish depending on patient preference. This study protocol was reviewed and approved by the institutional review board of MHS, Albert Einstein College of Medicine.

### Patient data

A database maintained by the YMCA had demographic information, information about participants’ primary care site, and the characteristics of sessions. De-identified data were made available by the YMCA to MHS staff for analysis. Demographic factors included age group (18–44, 45–59, and ≥60 y), preferred language (English, Spanish, other/missing), and sex (female, male, and missing).

Data on primary care sites included the number of referrals to the YMCA’s DPP made by each provider (<5, 5–19, and ≥20); type of referring site (teaching site [physician residents and medical students provide care with supervision from attending physicians] vs nonteaching site [attending physicians provide patient care]); season of referral (spring, summer, fall, or winter); primary season in which sessions were held (eg, a session starting in mid-February would be coded as spring because most sessions occurred during the spring); whether the primary care site was a Federally Qualified Health Center (FQHC); time between the referral of the patient and the start of the sessions (<2, 2–3, and ≥4 months); and time of day of the session (ie, weekday during the day, weekday during the evening, or Saturday).

The YMCA’s DPP categorized patients as “placed” if scheduled to attend a session or “never placed” if never scheduled to attend a session. Subsequently, placed patients were further categorized based on their attendance in YMCA’s DPP sessions. Patients who attended 3 or more sessions were categorized as “enrolled.” Patients who never attended any sessions or dropped out of the program before attending 3 sessions were categorized as “never enrolled.”

### Analysis

This study was a secondary data analysis of MHS patients referred and placed in YMCA’s DPP. The first analysis included 1,249 MHS patients referred between July 29, 2010, and November 12, 2015, who were placed in a YMCA DPP session or had an active referral on file as of February 1, 2014. An active referral indicated a patient who was evaluated and referred to YMCA’s DPP by a health care provider and an effort was made to recruit that patient. The initial analysis was conducted to identify whether any demographic factors or primary care site factors were associated with being placed versus never placed among referred patients and whether there were factors associated with being enrolled versus never enrolled among placed patients. We compared the proportion within each category to a predefined reference group by using logistic regression models.

The second analysis comprised 287 MHS patients referred to YMCA’s DPP who started attending sessions between October 26, 2011, and July 28, 2015, and had recorded weight-change data available. Some of the patients in this analysis (n = 89) were enrolled in YMCA’s DPP and had weight-change information but were missing information regarding their referral; these patients were excluded from the prior referral analysis. Baseline weight was obtained from the first session attended by the patient. Final weight came from the last session attended by the patient, which may not have been obtained after completing the 16 core sessions. Two outcome variables were evaluated: average percentage weight change, which was evaluated as a continuous variable; and patients who met the weight-loss targets of more than 5% of initial body weight or who did not meet the weight-loss targets, evaluated as a dichotomous outcome variable. Five percent was used as a cutoff because it is the established National DPP program weight-loss goal and is used as the metric for evaluation of nationally registered programs (10). The significance of continuous weight change by the factors described above was evaluated by using a pairwise *t* test comparing the mean percentage weight change in one group to a predefined reference group. The significance of the categorical weight-change variable was assessed by comparing the proportion within each category to a predefined reference group by using logistic regression models.

An α level of .05 was used to determine statistical significance, and an α level of .10 was considered marginally significant. We used Stata 14.1 for Windows (StataCorp LP) to perform all analyses.

## Results

Of the MHS patients referred to the YMCA’s DPP, 33.6% were placed. Among those placed, 47.1% attended 3 or more sessions. Among the patients who were referred to YMCA’s DPP, patients aged 18 to 44 years (72.9%) were least often placed, whereas approximately 36% of patients aged 45 to 59 years and patients aged 60 years or older were placed (Table 1). Patients preferring sessions in Spanish were less often placed than patients preferring English. No difference was observed in the placement of patients by sex, though patients missing sex information were somewhat less likely to be placed. A U-shaped relationship between number of referrals and placements was observed. Patients referred by providers making 20 or more referrals or providers making fewer than 5 referrals were least often placed, whereas patients referred by a provider making 5 to 19 referrals were most often placed. No difference in placement was observed by teaching versus nonteaching practice or whether the site was an FQHC. Fewer referrals were made in the summer, but these summer referrals were more often placed than winter referrals (41.0% vs 32.2%, *P* = .03).

**Table 1:** Baseline Comparisons of Montefiore Hospital System Patients in New York State YMCA’s Diabetes Prevention Program, 2010–2015

Characteristic	Total Referrals^a^, n	Among Referred (n = 1,249)			Among Those Placed (n = 420)		
		Never Placed,^b^ n (%)	Placed, n (%)	*P *Value^c^	Never Enrolled, n (%)	Enrolled^b^ n (%)	*P *Value^c^
**Total**	1,249	829 (66.4)	420 (33.6)	NA	222 (52.9)	198 (47.1)	NA
**Age at referral, y**							
18–44	350	255 (72.9)	95 (27.1)	.01	63 (66.3)	32 (33.7)	<.001
45–59	536	341 (63.6)	195 (36.4)	.98	105 (53.9)	90 (46.2)	.02
≥60	350	223 (63.7)	127 (36.3)	Reference	52 (40.9)	75 (59.1)	Reference
**Preferred language**							
English	879	568 (64.6)	311 (35.4)	Reference	155 (49.8)	156 (50.2)	Reference
Spanish	179	129 (72.1)	50 (27.9)	.06	27 (54.0)	23 (46.0)	.59
Other/missing	191	132 (69.1)	59 (30.9)	.24	40 (67.8)	19 (33.2)	.01
**Sex**							
Female	888	582 (65.5)	306 (34.5)	Reference	158 (51.6)	148 (48.4)	Reference
Male	217	142 (65.4)	75 (34.6)	.98	42 (56.0)	33 (44.0)	.50
Missing	144	105 (72.9)	39 (27.1)	.08	22 (56.4)	17 (43.6)	.57
**Referrals by each provider^d^**							
<5	294	194 (66.0)	100 (34.0)	.054	53 (53.0)	47 (47.0)	.81
5–19	317	172 (54.3)	145 (45.7)	<.001	72 (49.7)	73 (50.3)	.39
≥20	602	435 (72.3)	167 (27.7)	Reference	91 (54.5)	76 (45.5)	Reference
**Referring practice^e^**							
Nonteaching	700	461 (65.9)	239 (34.1)	Reference	121 (50.7)	118 (49.4)	Reference
Teaching	549	368 (67.0)	181 (33.0)	.66	101 (55.8)	80 (44.2)	.29
**Referral season**							
Spring	304	201 (66.1)	103 (33.9)	.62	62 (60.1)	41 (39.8)	.03
Summer	188	111 (59.0)	77 (41.0)	.03	42 (54.6)	35 (45.5)	.22
Fall	296	204 (68.9)	92 (31.1)	.75	50 (54.4)	42 (45.7)	.21
Winter	460	312 (67.8)	148 (32.2)	Reference	68 (46.0)	80 (54.1)	Reference
**Session season^f^**							
Spring	NA				73 (54.5)	61 (45.5)	Reference
Summer					66 (42.9)	88 (57.1)	.002
Fall					65 (63.1)	38 (36.9)	.049
Winter					18 (62.1)	11 (37.9)	.06
**Referral from Federally Qualified Health Center**							
Yes	585	381 (65.1)	204 (34.9)	Reference	107 (52.5)	97 (47.6)	Reference
No	663	447 (67.4)	216 (32.6)	.39	115 (53.2)	101 (46.8)	.87
**Time between referral and first scheduled session**							
<2 months	NA				123 (45.6)	147 (54.4)	Reference
2 to <4 months					41 (54.0)	35 (46.1)	.20
≥4 months					58 (78.4)	16 (21.6)	<.001

Abbreviation: NA, not applicable.

a Patients referred from July 29, 2010, through November 12, 2015, who had been placed in a YMCA Diabetes Prevention Program session or had an active referral on file as of February 1, 2014. Data may not sum to the total because of missing data.

b Never placed refers to patients who were referred to the program but never enrolled in a session. Enrolled patients attended 3 or more sessions, and never enrolled patients attended fewer than 3 sessions.

c *P* values estimated using logistic regression.

d Number of referrals made by the referring provider.

e A teaching site was one in which physician residents and medical students provide care with supervision from attending physicians. A nonteaching site was one in which attending physicians provided patient care.

f Based on the season in which the 16-week session was predominantly held. For example, a session starting in mid-February would be coded as spring since most the session occurred during the spring as opposed to the winter.

Fifty-three percent of placed patients were never enrolled (Table 1). There was an age gradient in enrollment, with youngest (18–44 y) least often, and the oldest (≥60 y) most often enrolled. The proportion of enrolled was similar among patients preferring English versus Spanish, although patients preferring other languages or with missing language information were least often enrolled. Men and women were equally enrolled. Although the number of referrals made by each provider was associated with placement, no association was observed for number of referrals made by each provider and being enrolled. Type of practice or whether the primary care site was an FQHC was not associated with being enrolled. Patients referred in the spring were less often enrolled than patients referred in the winter. Patients who started their sessions within 2 months of their referral date were more often enrolled (54.4%) than patients who had to wait 4 or more months (21.6%). Lastly, patient enrollment was highest if the sessions took place in the summer (57.1%) and lowest in the fall (36.9%).

Among the 287 patients enrolled, the average weight change was −3.4% (Table 2). Approximately 30% of patients enrolled lost 5% or more of their body weight (Figure). Patients aged 45 to 59 years lost marginally less weight than those aged 60 years or older (−3.1% vs −3.8%; *P* = .07). No differences in weight-loss percentage were observed by sex. Patients enrolled in the fall and winter sessions lost the least weight (−2.8% and −2.3%, respectively). No other examined factor was associated with differences in weight loss.

**Table 2:** Weight Change and Proportion Losing Weight or With Stable Weight, by Selected Characteristics, Patients in Montefiore Hospital System in New York State YMCA’s Diabetes Prevention Program, 2011–2015^a^

Characteristic	n	Weight Change, %		Meeting Weight Loss Targets		
		Mean (SE)	*P* Value^b^	Did Not Meet Target for Weight Loss (<5% Weight Loss), n (%)	Met Target for Weight Loss (≥5% Weight Loss), n (%)	*P* Value^b^
**Total**	287	−3.4 (0.2)	NA	203 (70.7)	84 (29.3)	NA
**Age at referral, y**						
<45	51	−3.5 (0.4)	.60	35 (68.6)	16 (31.4)	.84
45–59	127	−3.1 (0.3)	.07	94 (74.0)	33 (26.0)	.24
≥60	106	−3.8 (0.3)	Reference	71 (67.0)	35 (33.0)	Reference
**Preferred language**						
English	239	−3.4 (0.2)	Reference	168 (70.3)	71 (29.7)	Reference
Spanish	27	−3.8 (0.6)	.46	20 (74.1)	7 (25.9)	.68
Other/missing	21	−3.0 (0.8)	.56	15 (71.4)	6 (28.6)	.91
**Sex**						
Female	210	−3.4 (0.2)	Reference	146 (69.5)	64 (30.5)	Reference
Male	46	−3.5 (0.4)	.85	35 (76.1)	11 (23.9)	.38
Missing	31	−3.2 (0.6)	.69	22 (71.0)	9 (29.0)	.87
**Referrals by each provider**						
<5	55	−3.7 (0.4)	.72	39 (70.9)	16 (29.1)	.85
5–19	90	−3.1 (0.3)	.34	60 (66.7)	30 (33.3)	.37
≥20	137	−3.6 (0.2)	Reference	99 (72.3)	38 (27.7)	Reference
**Referral placement rate^c^, %**						
<20	32	−3.8 (0.5)	.64	19 (59.4)	13 (40.6)	.39
20–29.9	105	−3.6 (0.3)	Reference	75 (71.4)	30 (28.6)	Reference
30–49.9	63	−3.3 (0.4)	.57	43 (68.3)	20 (31.8)	.66
≥50	82	−3.2 (0.3)	.45	61 (74.4)	21 (25.6)	.42
**Referring practice^d^**						
Nonteaching	166	−3.3 (0.2)	Reference	123 (74.1)	43 (25.9)	Reference
Teaching	121	−3.6 (0.3)	.39	80 (66.1)	41 (33.9)	.14
**Referral season**						
Spring	49	−4.2 (0.5)	.14	31 (63.3)	18 (36.7)	.43
Summer	56	−2.9 (0.3)	.18	44 (78.6)	12 (21.4)	.22
Fall	67	−3.2 (0.4)	.47	48 (71.6)	19 (28.4)	.78
Winter	115	−3.5 (0.3)	Reference	80 (69.6)	35 (30.4)	Reference
**Session season^e^**						
Spring	130	−4.1 (0.3)	Reference	79 (60.8)	51 (39.2)	Reference
Summer	48	−3.0 (0.4)	.03	37 (77.1)	11 (22.9)	.045
Fall	91	−2.8 (0.3)	.002	72 (79.1)	19 (20.9)	.004
Winter	18	−2.3 (0.6)	.01	15 (83.3)	3 (16.7)	.08
**Referral from Federally Qualified Health Center**						
Yes	141	−3.6 (0.3)	Reference	94 (66.7)	47 (33.3)	Reference
No	146	−3.2 (0.2)	.32	109 (74.7)	37 (25.3)	.14
**Time between referral and first scheduled session**						
<2 months	223	−3.5 (0.2)	Reference	157 (70.4)	66 (29.6)	Reference
2–<4 months	40	−3.0 (0.4)	.31	30 (75.0)	10 (25.0)	.55
≥4 months	24	−3.6 (0.6)	.90	16 (66.7)	8 (33.3)	.70
**Start year**						
2011–2012^f^	79	−3.6 (0.3)	.58	53 (67.1)	26 (32.9)	.40
2013	71	−3.4 (0.4)	.85	49 (69.0)	22 (31.0)	.57
2014	62	−3.3 (0.4)	.95	46 (74.2)	16 (25.8)	.91
2015	75	−3.3 (0.3)	Reference	55 (73.3)	20 (26.7)	Reference
**Session time**						
Weekday, working hours	88	−3.4 (0.3)	Reference	67 (76.1)	21 (23.9)	Reference
Weekday, evening hours^g^	155	−3.5 (0.2)	.86	105 (67.7)	50 (32.3)	.17
Saturday	44	−3.1 (0.4)	.54	31 (70.5)	13 (29.6)	.73

Abbreviation: NA, not applicable; SE, standard error.

a Data may not sum to the total because of missing values.

b *P* values comparing mean weights were estimated by using a *t* test with the reference group. *P* values for percentage differences were estimated by using logistic regression.

c Proportion of patients referred by a provider who were placed into the program. For example, a provider referring 40 patients, with 10 placed, would have a referral placement rate of 25%.

d A teaching site was one in which physician residents and medical students provided care with supervision from attending physicians. A nonteaching site was one in which attending physicians provided patient care.

e Based on the season in which the 16-week session was predominantly held. For example, a session starting in mid-February would be coded as “spring” because most the session occurred during the spring as opposed to the winter.

f A small number of participants started in 2011 (<10), so the data were collapsed to increase statistical stability.

g Defined as starting at or after 4:30 PM. Eighty-five percent of evening classes started between 5:30 PM and 6:30 PM.

**Figure:**
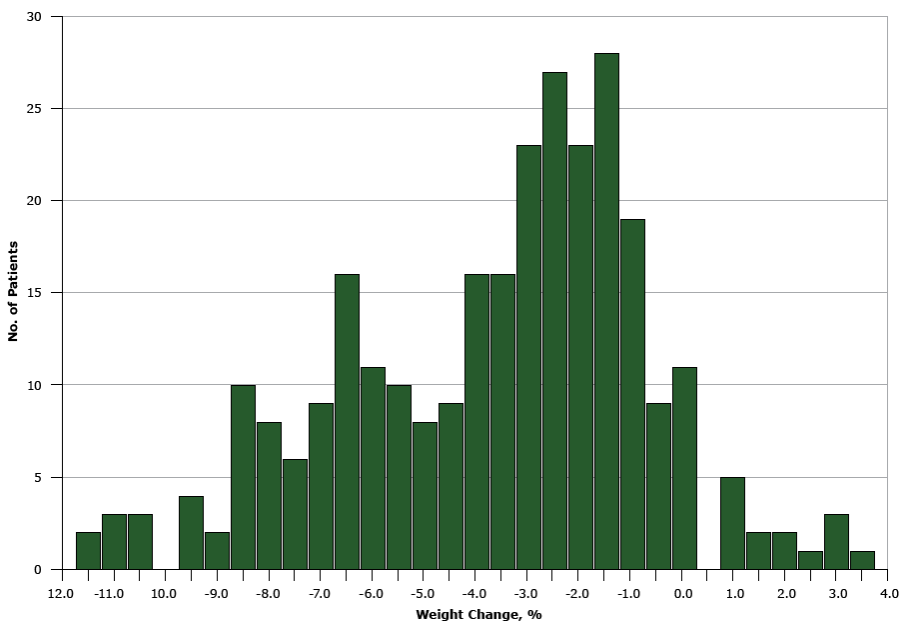
Distribution of weight change among 287 Montefiore Health System patients enrolled in the New York State YMCA’s Diabetes Prevention Program, 2011–2015. **Table:** 

Weight Change, %	No. of Patients
−11.5	2
–11.0	3
–10.5	3
–10.0	0
–9.5	4
–9.0	2
–8.5	10
–8.0	8
–7.5	6
–7.0	9
–6.5	16
–6.0	11
–5.5	10
–5.0	8
–4.5	9
–4.0	16
–3.5	16
–3.0	23
–2.5	27
–2.0	23
–1.5	28
–1.0	19
–0.5	9
0.0	11
0.5	0
1.0	5
1.5	2
2.0	2
2.5	1
3.0	3
3.5	1

## Discussion

### Placement and enrollment

CDC has developed guidelines to facilitate referral and enrollment of patients from the primary care setting into intensive lifestyle interventions such as YMCA’s DPP (10). However, evaluations of the process for placing patients in YMCA’s DPP are lacking. In our study, a third of the patients referred were placed in the program. Older patients and patients preferring sessions in English were most often placed. Understanding the importance of communicating and offering health behavior programs in Spanish and other languages is an important part of implementing DPPs. Tailoring the DPP to literacy needs and cultural preferences is an important component to successfully implementing DPPs in Spanish-speaking communities (11,12). Demographic differences in placement could also reflect an inability to reach some patients (eg, patients with missing or nonworking telephone number) or challenges in providing an adequate number of sessions in Spanish, for example. One unexpected finding was that patients referred by a provider making 20 or more referrals were less often placed than patients referred by providers who made fewer referrals. Whether this is a chance finding requires additional evaluation of other data, but it may suggest that providers making a large number of referrals are less selective in referring patients who are interested or able to participate in the program.

Although the National DPP defines enrollment as attending 4 or more sessions in the first 4 to 6 months of starting the program (10), the YMCA of the USA and the Diabetes Prevention and Control Alliance defines enrollment as attending 3 or more sessions. Therefore, YMCA’s DPP reporting includes participant outcomes for those attending 3 or more sessions. Comparing enrollment rates across community-based DPP studies is difficult because many studies do not report this information, the criteria for enrollment may differ from that of the National DPP, or the populations served are different across study sites. Nevertheless, a review of DPP enrollment rates across 16 studies by Whittemore reported enrollments between 57% and 96% (13). In our study, 47.1% of patients placed in YMCA’s DPP attended 3 or more sessions.

We showed that patients were less often enrolled if they waited longer between referral and start of first session. Numerous factors may influence this lag time. Patient-level factors such as being difficult to reach or having an inflexible or unpredictable schedule may hamper prompt placement. Systems-level factors, including number of sessions offered and their times and locations, may also influence the ability to place patients quickly. Because sessions in the current program were offered on an ad hoc basis, it is not possible to determine which factors influenced the lag time. We also showed that patients aged 60 years or older were more often enrolled than younger adults. This finding is consistent with weight-loss studies where retention rates were greatest among older participants (14–16). The reasons often provided to account for the high attrition among younger individuals include less financial stability, greater childcare burden, and less flexible work schedules (17). Attrition among younger participants is of particular concern given that approximately 26% of adults aged less than 60 years in the United States have prediabetes (18). Also, patients were more often placed if they were referred during the summer than the winter and less often enrolled if referred during the spring than the winter. Enrollment and weight loss were highest among those attending sessions in the spring and the summer. The seasonal relationships observed may reflect the times of year when patients prioritize weight-loss behaviors such as physical activity (19,20).

Although our patients requesting sessions in Spanish were less often placed in YMCA’s DPP (*P* = .06), we observed that they were enrolled in the program just as often compared with patients requesting English. This suggests that language preference is associated with initial engagement in the program but has little effect once patients are enrolled, indicating that the program was effective at engaging Spanish-speaking patients once enrolled. The National DPP is dedicated to providing the program to all communities and offers materials in many different languages. This is an important consideration in the Bronx, where 55% are of Hispanic/Latino origin and 47% speak Spanish at home (21).

### Weight loss

MHS serves a predominantly low-income and medically underserved population. Many challenges in these communities may influence weight-loss programming, including high levels of poverty and limited access to healthy food options. The average weight loss in our study was 3.4%, which is less than the National DPP goal of 5% average weight loss. Because of the uncertainty of the dates of the data collection, the weight change cannot be categorized as occurring over a specific time period (eg, 16-week weight change), although the longest possible length of time our patients’ weights were recorded was 4 months. Nevertheless, in our study, 30% of patients lost 5% or more of their body weight during their enrollment. Reports from the New York State YMCA’s DPP show that 40% of participants lost 5% or more of their initial body weight (6).

### Limitations

This study was a secondary analysis of routinely collected data. Numerous variables that would have been of interest were unavailable, including data on weekly participation (eg, determining exactly when patients dropped out), weights for every session (eg, determining when weight loss is most likely to occur), the patient’s address (eg, does distance to location where DPP is offered affect attendance), and data on race/ethnicity of participants. In addition, qualitative data on reasons for dropping out or not being placed would add value, but were not available. The data presented here comprised only MHS patients, who may have been placed in sessions with non-MHS patients. Therefore, the results may not be generalizable to all participants in the YMCA’s DPP.

### Conclusions

The results of this study have implications for the partnership of hospital systems and community-based organizations implementing DPP. Our findings indicate that approximately 16% of patients referred to YMCA’s DPP from our hospital system enrolled. More work is needed to increase the enrollment of patients referred to YMCA’s DPP. The results of our study point to a few areas where changes can occur to address placement and enrollment of patients but also point to where more information is necessary to further understand barriers. Lag time between referral and the start of the sessions needs to be reduced to maximize the likelihood of enrollment. The timing of referrals and sessions are important considerations when planning sessions. To optimize enrollment, efforts should be made to coordinate when referrals and sessions take place. Furthermore, efforts to target younger and Spanish-speaking adults are important.

An area in need of further research is that of the health care provider’s role in the identification and referral of eligible patients to YMCA’s DPP. We noticed that the number of referrals that a provider makes is associated with whether or not the patient was placed. These results highlight a need to better understand the process of patient selection and subsequent referral of patients during the clinical encounter. Incorporating referrals into the EHR is one strategy that has promise for increasing patient referrals to YMCA’s DPP (22).
